# Supplementary material for: Inactivation of TGFβ receptors in stem cells drives cutaneous squamous cell carcinoma
Source: Nat Commun. 2016 Aug 25;7:12493. doi: 10.1038/ncomms12493 (PMC5007296; doi:10.1038/ncomms12493)
Supplement: Supplementary Information — Supplementary Figures 1-15 and Supplementary Tables 1-4. [file ncomms12493-s1.pdf]

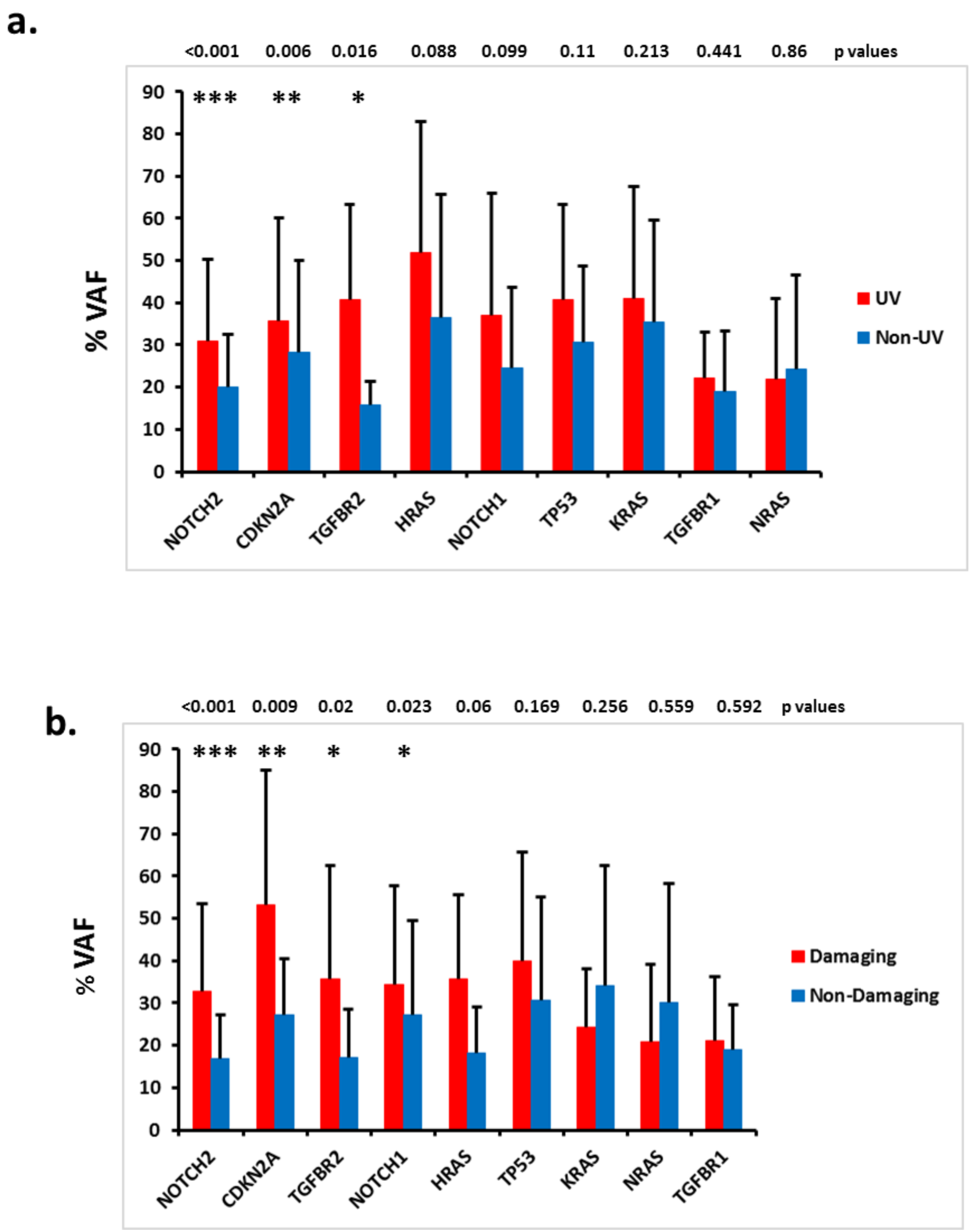

Supplementary Figure 1. Comapartive analysis of VAF/UV signature and predicted mutational consequence. (**a**) %VAF of mutations in the nine driver genes indicated separated into UV *versus* non-UV signature as indicated. (**b**) %VAF of mutations in the nine driver genes indicated separated into Damaging or Non-damaging, as determined by mutational significance prediction programmes (see Methods). error bars represent +/- s.e.m. and P values are shown with * referring to statistical significance throughout (*** p<0.001, ** p<0.01, * P<0.05, 2 tailed Students *t*-test). Data calculations are shown in Supplementary Data 14 and 25.

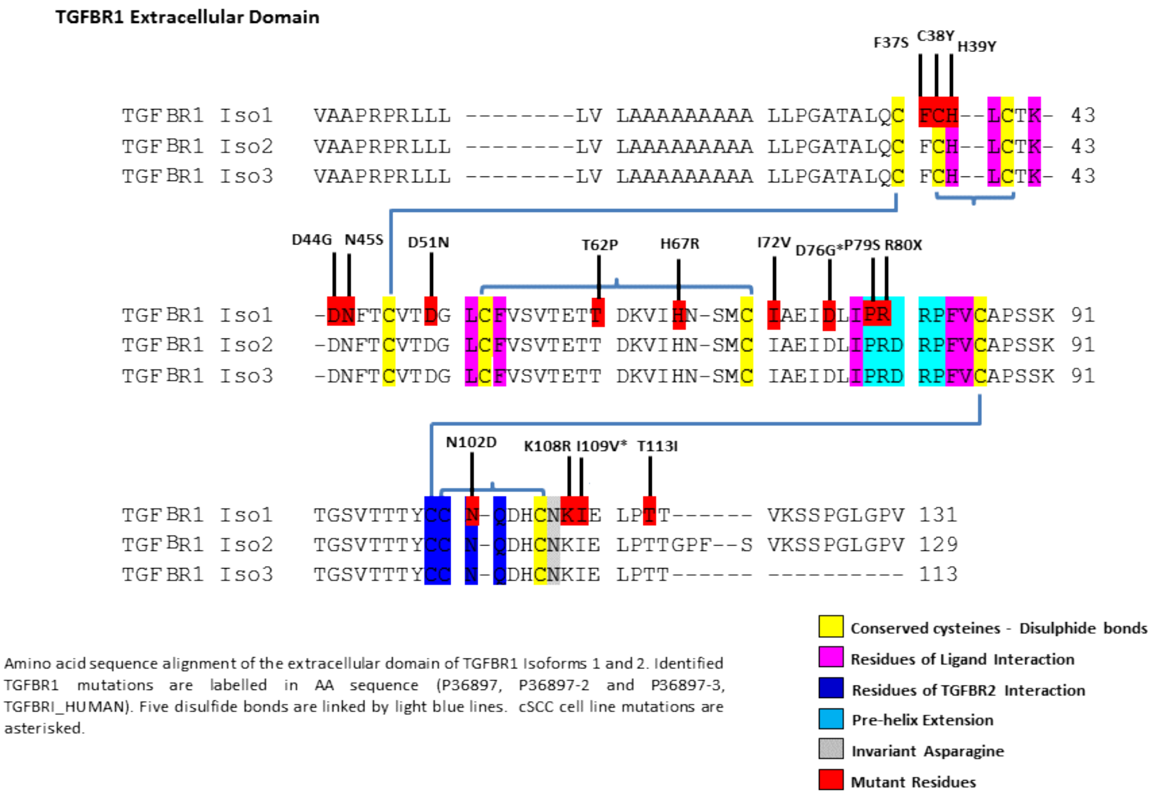

Supplementary Figure 2. Amino acid sequence alignment of the extracellular domain of TGFBR1 Isoforms. Identified TGFBR1 SNP's are labelled in AA sequence (using UniProtKB codes: P36897, P36897-2 and P36897-3, TGFBRI_HUMAN). Five disulphide bonds are linked by light blue lines. cSCC cell line mutations are asterisked.

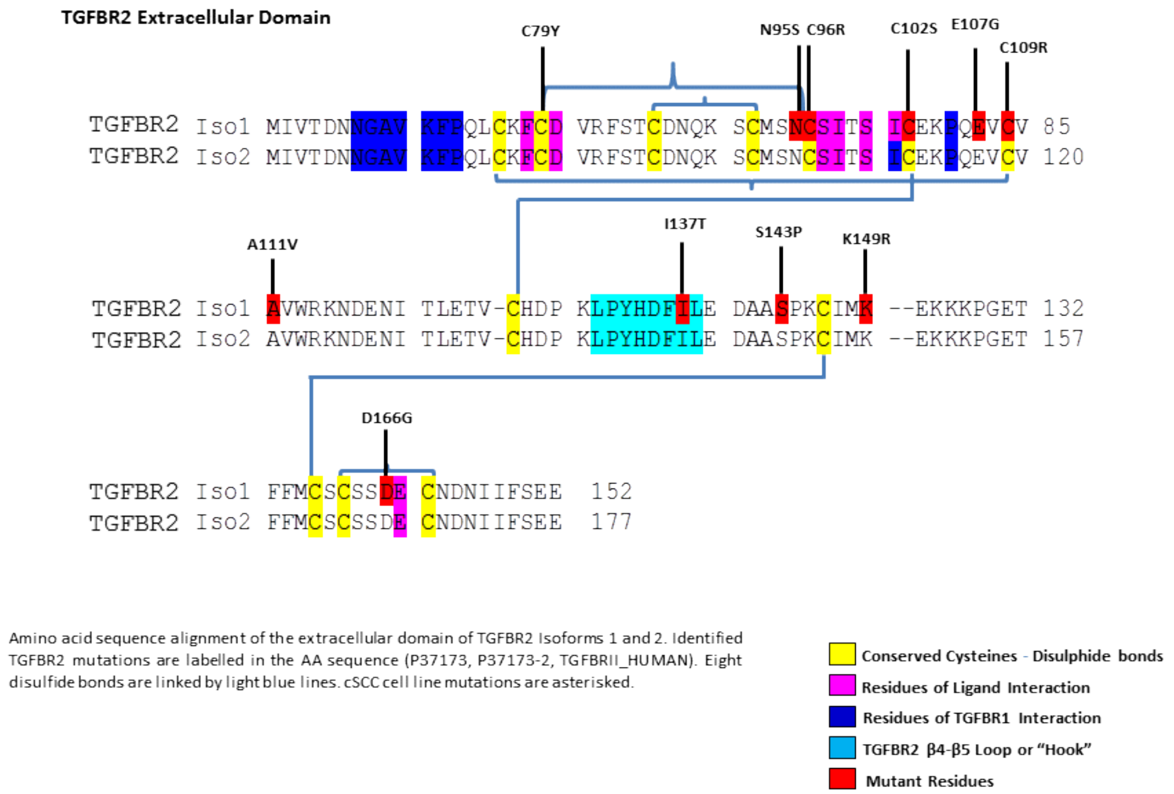

Supplementary Figure 3. Amino acid sequence alignment of the extracellular domain of TGFBR2 Isoforms. Identified TGFBR2 SNP's are labelled in AA sequence (using UniProtKB codes: P37173, P37173-2, TGFBRII_HUMAN). Eight disulphide bonds are linked by light blue lines. cSCC cell line mutations are asterisked.

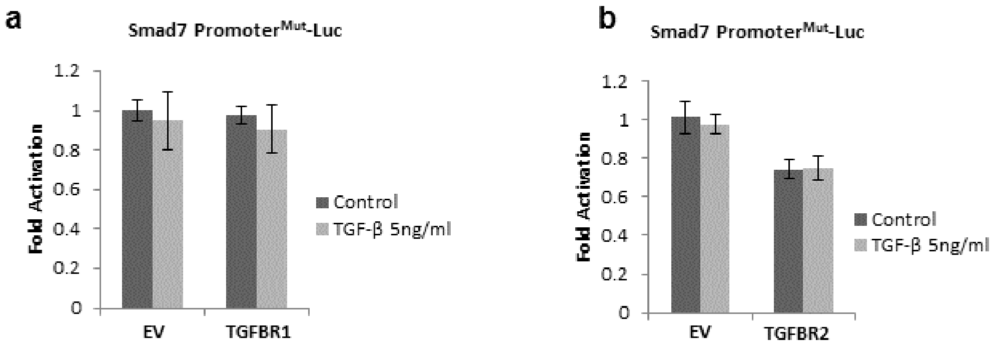

Supplementary Figure 4. TGFβ receptor stimulated Smad7 reporter activity requires Smad binding sites. (Related to Figure 4). (**a**) Indicated TGFBR1 plasmids were co-transfected into TGFBR1-null MEFs and assayed for Smad7-Promoter Mutant Luciferase (Smad7 Promoter^{mut}-Luc, has SMAD binding site mutated) reporter gene activity with and without TGFβ stimulation. EV is empty vector control, WT is wild type. (**b**) Indicated TGFBR2 plasmids were co-transfected into TGFBR2-null T47D cells and assayed for Smad7-Mutant Luciferase (Smad7mut-Luc) reporter gene activity. EV is empty vector control, WT is wild type. All data represents mean +/- s.d., n=3.

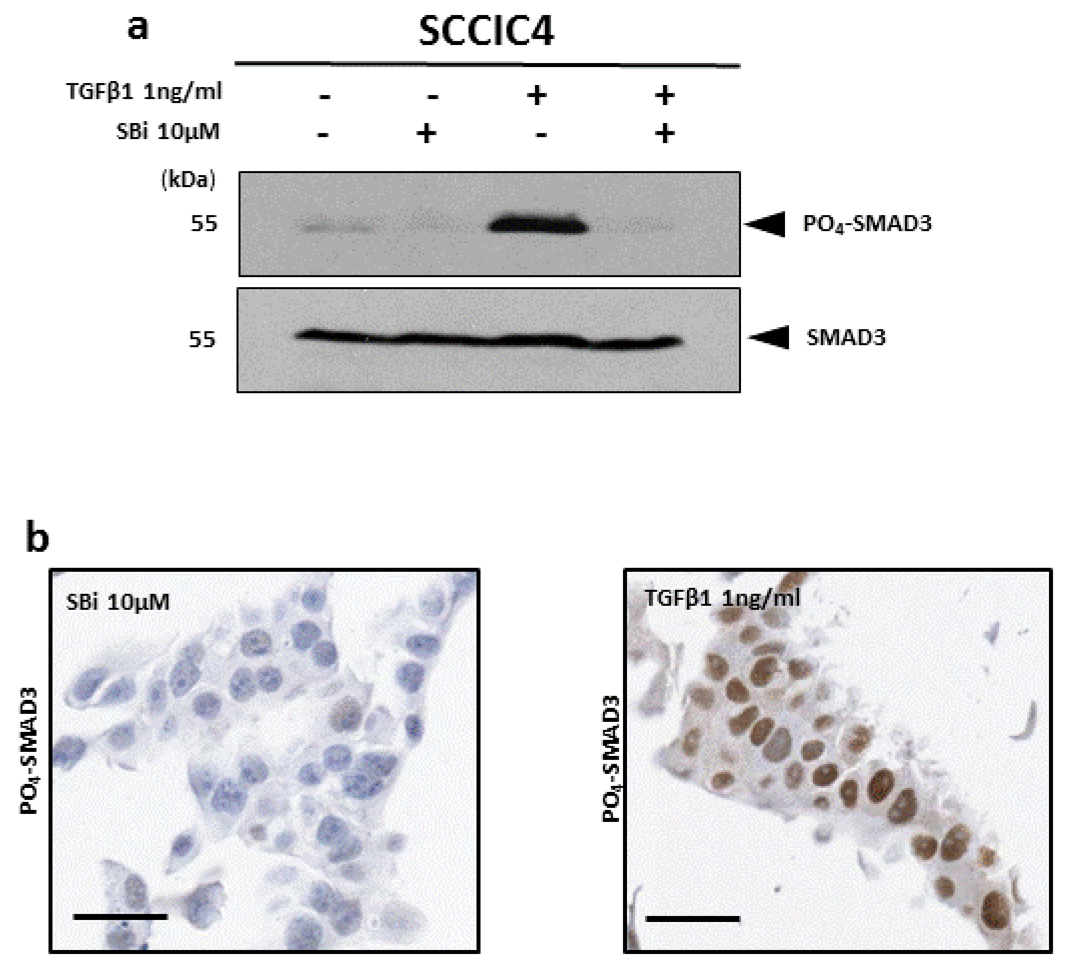

Supplementary Figure 5. **Optimisation of PO_4-SMAD3 antibody for IHC (Related to Figure 4).** SCCIC4 cells were treated with vehicle control (4mM HCl, 1mg/Ml BSA; DMSO, 1:1000 dilution), 1ng/ml TGFβ1 for 1 hour with or without pre-treatment with 10 μM TGFβR1 kinase inhibitor SB-431542 (Sbi) as indicated. (**a**) Cells were lysed in 4xSDS sample buffer and analysed by western blotting for anti-PO_4-SMAD3 and SMAD3 antibodies as indicated. Blots displayed are representative scans of at least 2 separate experiments. (**b**) Parallel samples were formalin fixed, pelleted into agarose and embedded into paraffin wax for IHC. Representative images of treated cells as indicated taken at x200 magnification on Aperio Imagescope. Scale bar, 50 μM. Representative images are shown showing enhancement of nuclear signal with TGFβ stimulation and loss of signal with SB-431542 treatment.

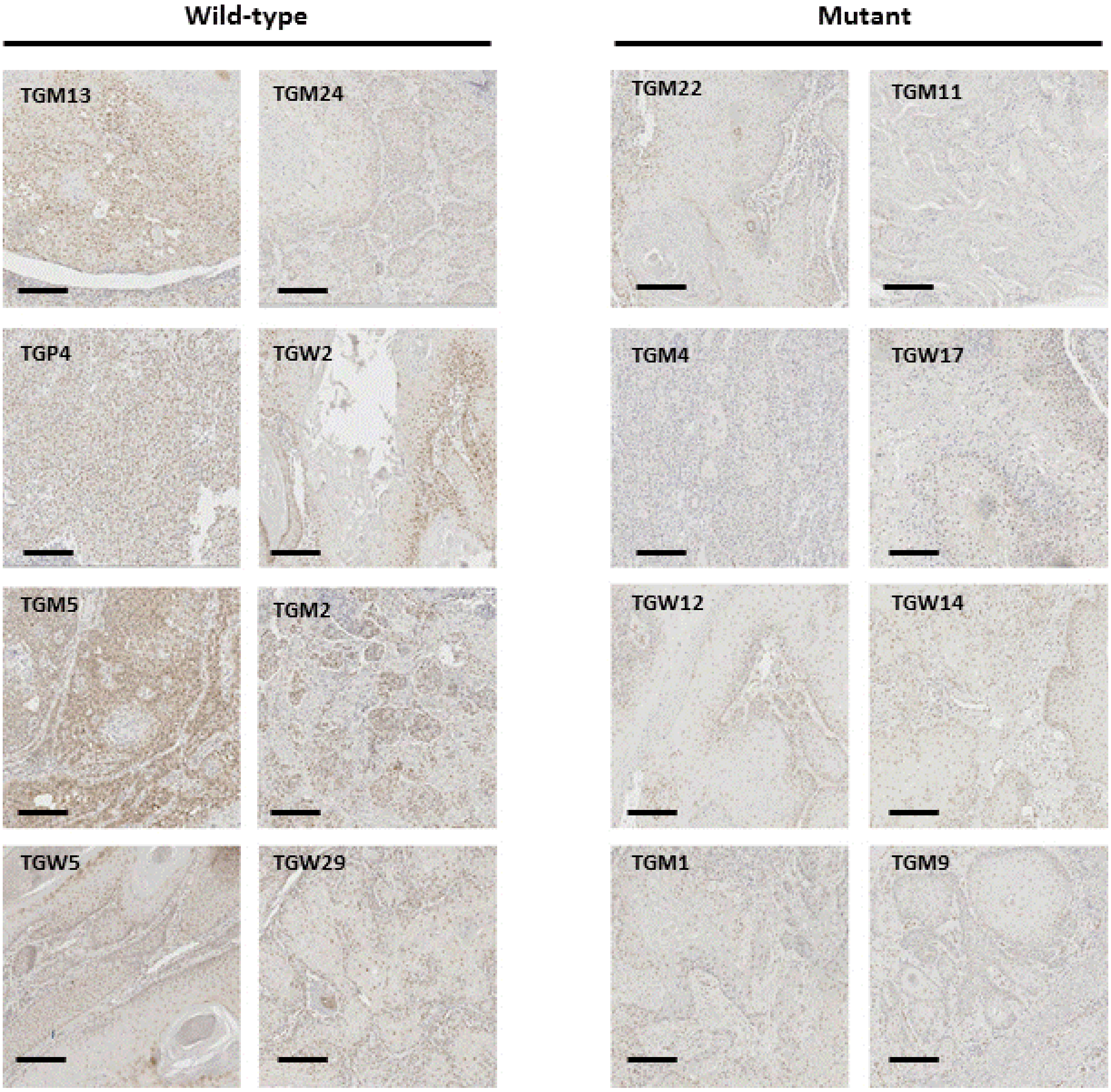

Supplementary Figure 6. Endogenous PO_4-SMAD3 activity is reduced in TGFβ receptor mutant tumours (Related to Figure 4). PO_4-SMAD3 activity was assessed by IHC in wild type and mutant tumors (n=8) and assessed by histoscore. Representative images of TGFβ receptor wild type tumors (left) and TGFβ receptor mutant tumors (right) are shown. Scale bar, 100 μM.

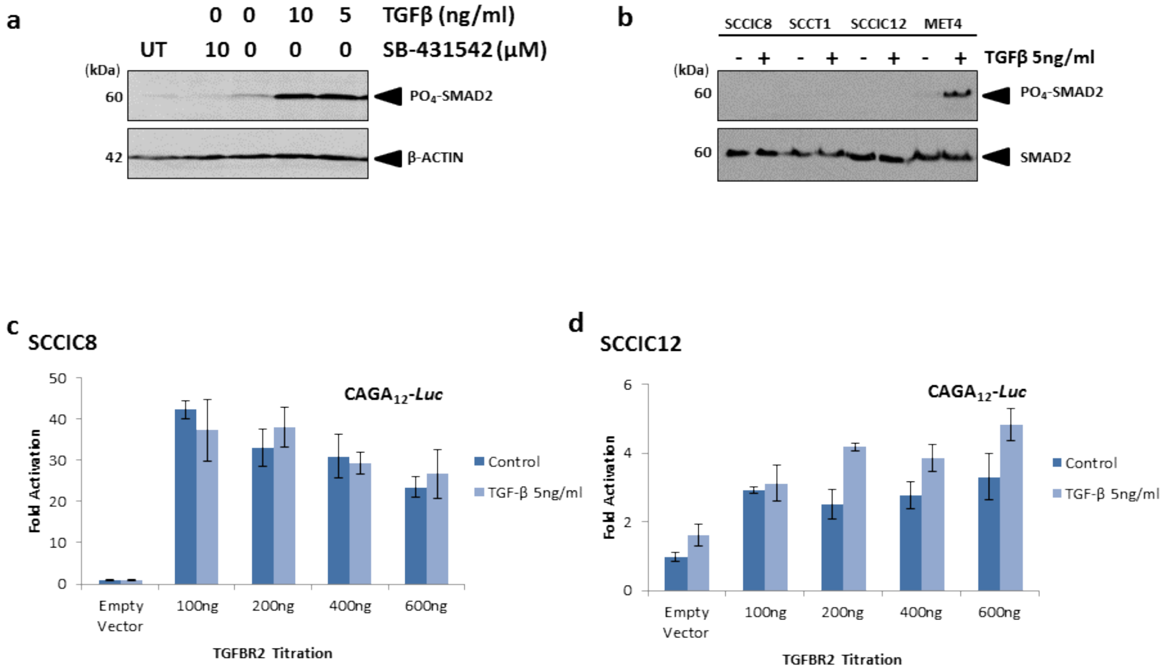

Supplementary Figure 7. TGFBR2 mutant cell lines fail to activate PO_4-SMAD activity which wild type TGFBR2 restores. (**Related to Figure 4).** (**a**) Western Blot of PO_4-SMAD2 activity in response to exogenous TGFβ stimulation and inhibition. NHK's were induced with indicated treatments for 1 hour (UT = Untreated). Western Blot was performed on lysates using indicated antibodies. β-ACTIN as loading control. Protein band sizes as indicated. Blots shown are representative scans of at least 3 separate experiments. (**b**) Western blots of PO_4-SMAD2 expression in response to 1 hour treatment of either vehicle control (4mM HCl, 1mg/mL BSA 1:1000 dilution) or 5ng/mL TGFβ in SCCIC8, SCCT1 and SCCIC12 (TGFBR2 mutant) and MET4 (TGFBR2 wild-type) cSCC cell lines. Protein band sizes as indicated. Blots shown are representative scans of at least 3 separate experiments. (**c**) Transfection of wild type TGFBR2 restores $CAGA_{12}$-Luciferase reporter activity in TGFBR2-null SCCIC8 cell lines. (**d**) Transfection of wild type TGFBR2 restores $CAGA_{12}$-Luciferase reporter activity in TGFBR2-null SCCIC12 cell lines. For (**c-d**): Cells were co-transfected with $CAGA_{12}$-Luciferase and increasing concentrations of wild type TGFBR2 expression plasmid as indicated. Luciferase activity was measured 24 hours after transfection with and without TGFβ1 (5ng/ml) treatment for 4 hours as indicated. All data and error bars represent +/- s.e.m., n=3.

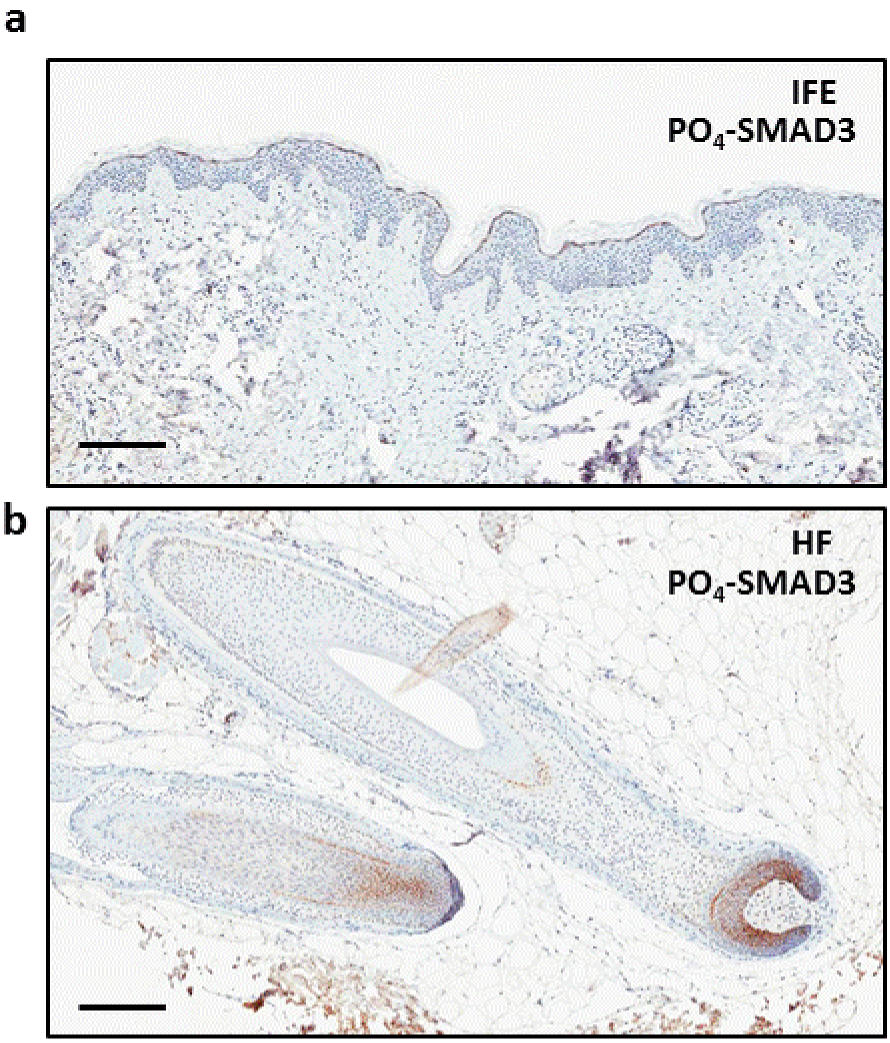

Supplementary Figure 8: Endogenous TGFβ signalling localised to the hair follicle matrix of normal human skin. Representative images demonstrating IHC of normal human scalp skin stained with anti-PO_4-SMAD3 antibody. (**a**) IFE = Interfollicular epidermis. (**b**) HF = Transverse sections through hair follicles within dermis. Scale bar, 200 µm.

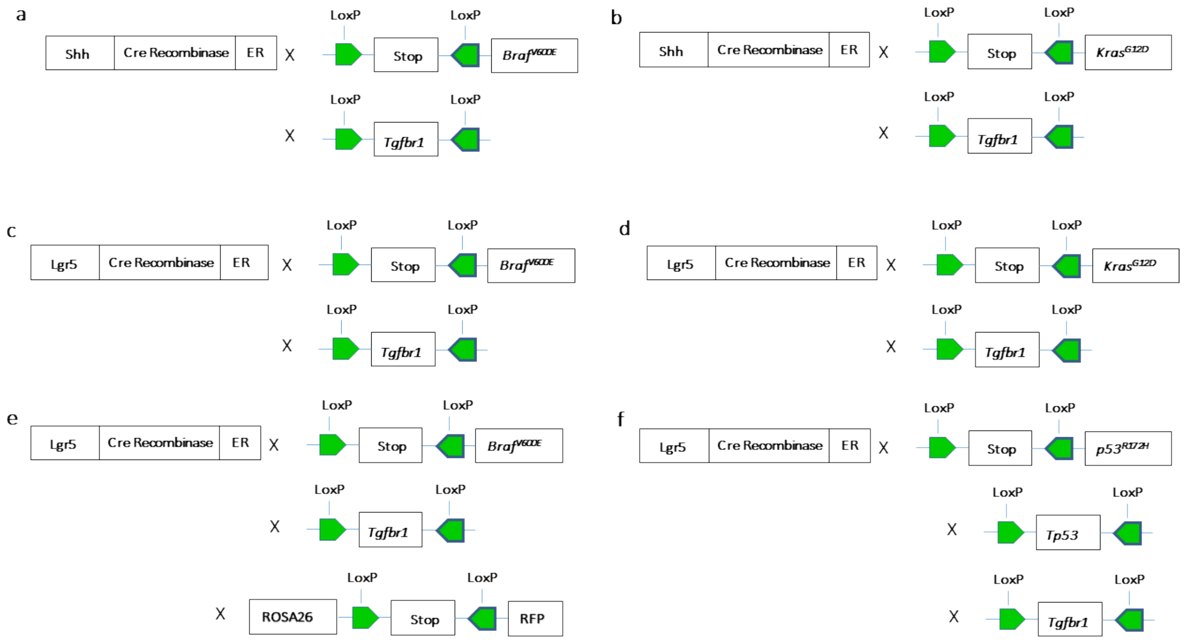

Supplementary Figure 9. **Mouse models schemes** (**a**) Targeting of *Braf*^{V600E} to SHH^{+ve} matrix cells. (**b**) Targeting of *Kras*^{G12D} to SHH^{+ve} matrix cells. (**c**) Targeting of *Braf*^{V600E} to Lgr5^{+ve} stem cells. (**d**) Lineage tracing scheme using *Rosa26-LSL-RFP*. (**e**) Targeting of *Kras*^{G12D} to LGR5^{+ve} stem cells. (**f**) Targeting of *Tp53*^{R172H} or deletion to LGR5^{+ve} stem cells. Cre recombinase ER refers to Tamoxifen regulated Cre recombinase. All models have the addition of targeted deletion of *Tgfbr1* as shown.

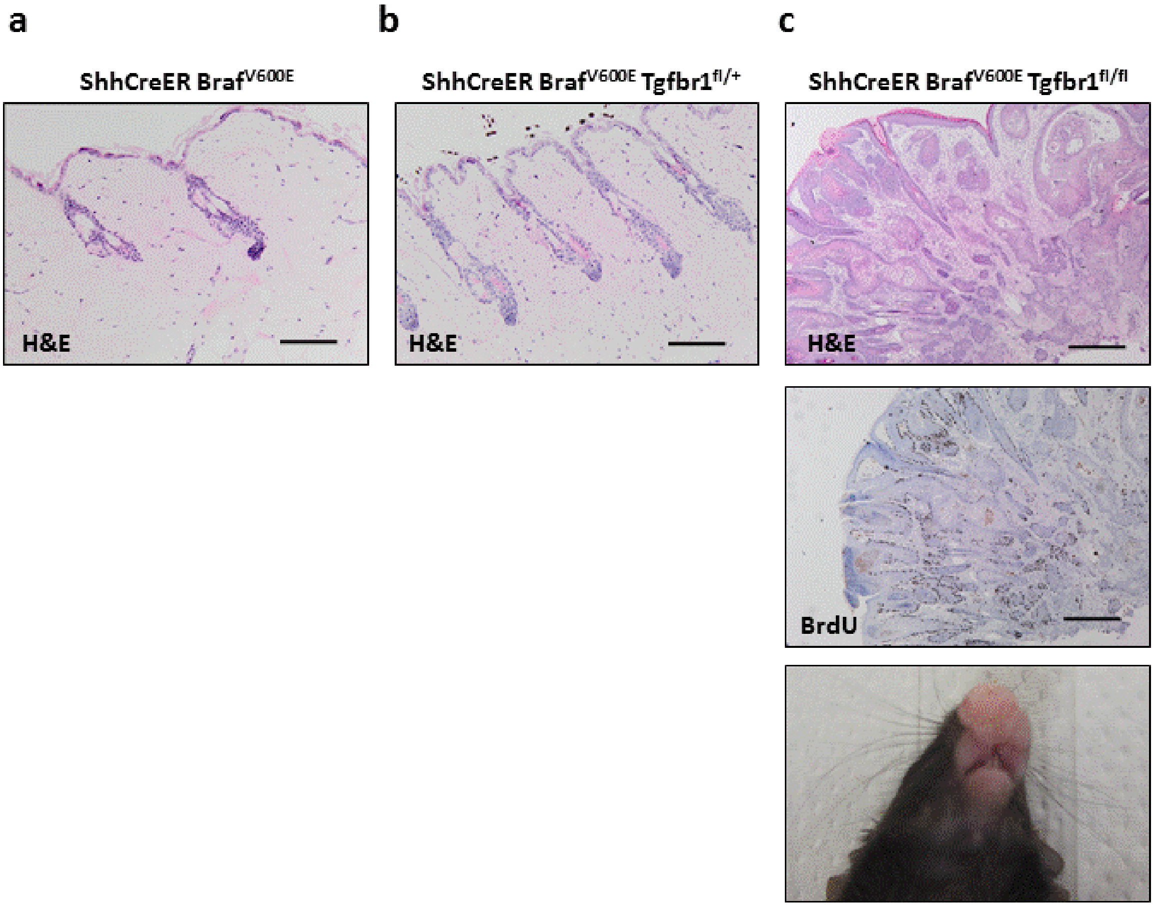

Supplementary Figure 10. ***Tgfbr1* loss coupled with *Braf*^{V600E} activation in SHH^{+ve} cells does not allow cSCC formation. (Related to Figure 5).** Representative H&E of skin from (**a**) *ShhCre*^{ER} *Braf*^{V600E} and (**b**) *ShhCre*^{ER} *Braf*^{V600E} *Tgfbr1*$^{fl/+}$ mice respectively at 230 and 196 days post Tamoxifen administration. Scale bar, 100 µm. (**c**) Macroscopic pictures of skin tumor, H&E and BrdU staining of *ShhCre*^{ER} *Braf*^{V600E} *Tgfbr1*$^{fl/fl}$ papilloma. Scale bar, 100 µm.

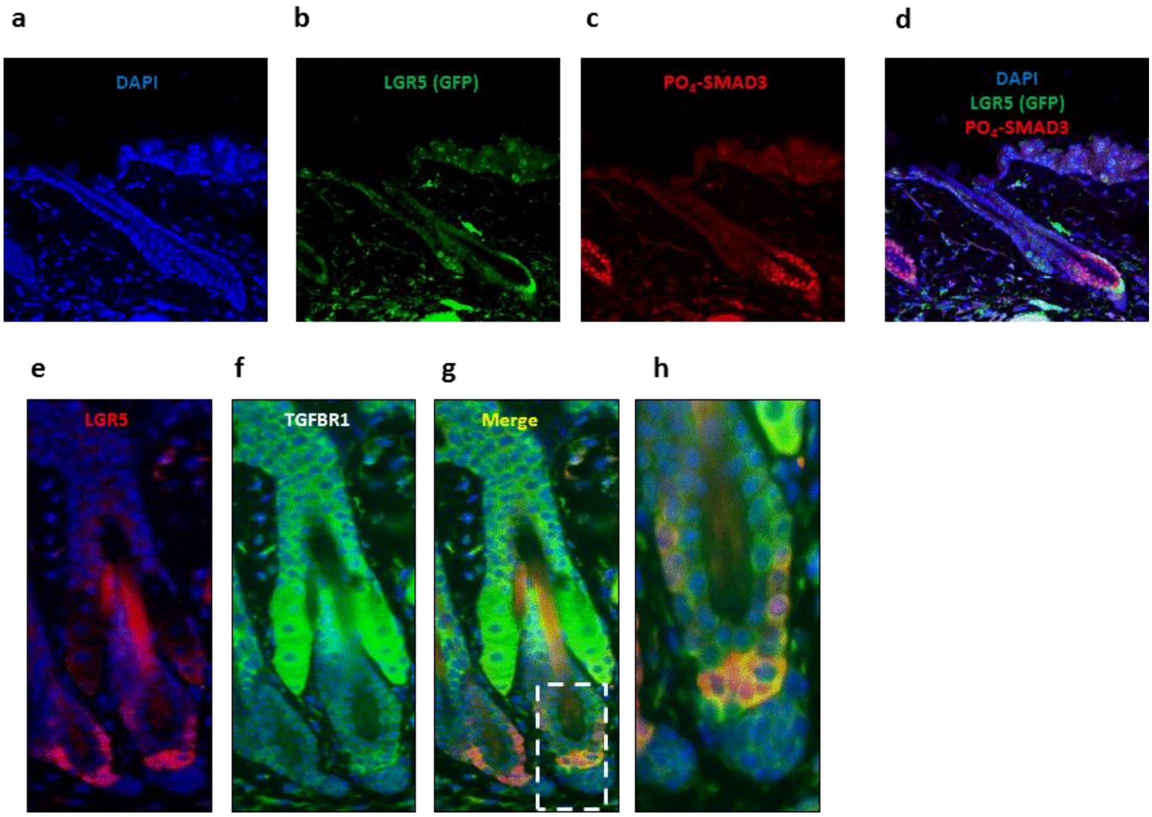

Supplementary Figure 11. **LGR5^{+ve} cells are highly enriched for both PO$_4$-SMAD3 and TGFBR1**. (**Related to Figure 6).** IF analysis of (**a**) DAPI, (**b**) LGR5-GFP and (**c**) PO$_4$-SMAD3 and (**d**) merge in telogen skin from Lgr5CRE^{ER} mice. IF analysis of (**e**) LGR5-GFP and (**f**) TGFBR1 in telogen skin from Lgr5Cre^{ER} mice. Merge is shown in (**g**) with blown up of boxed region shown in (**h**). Nuclei are counterstained with DAPI.

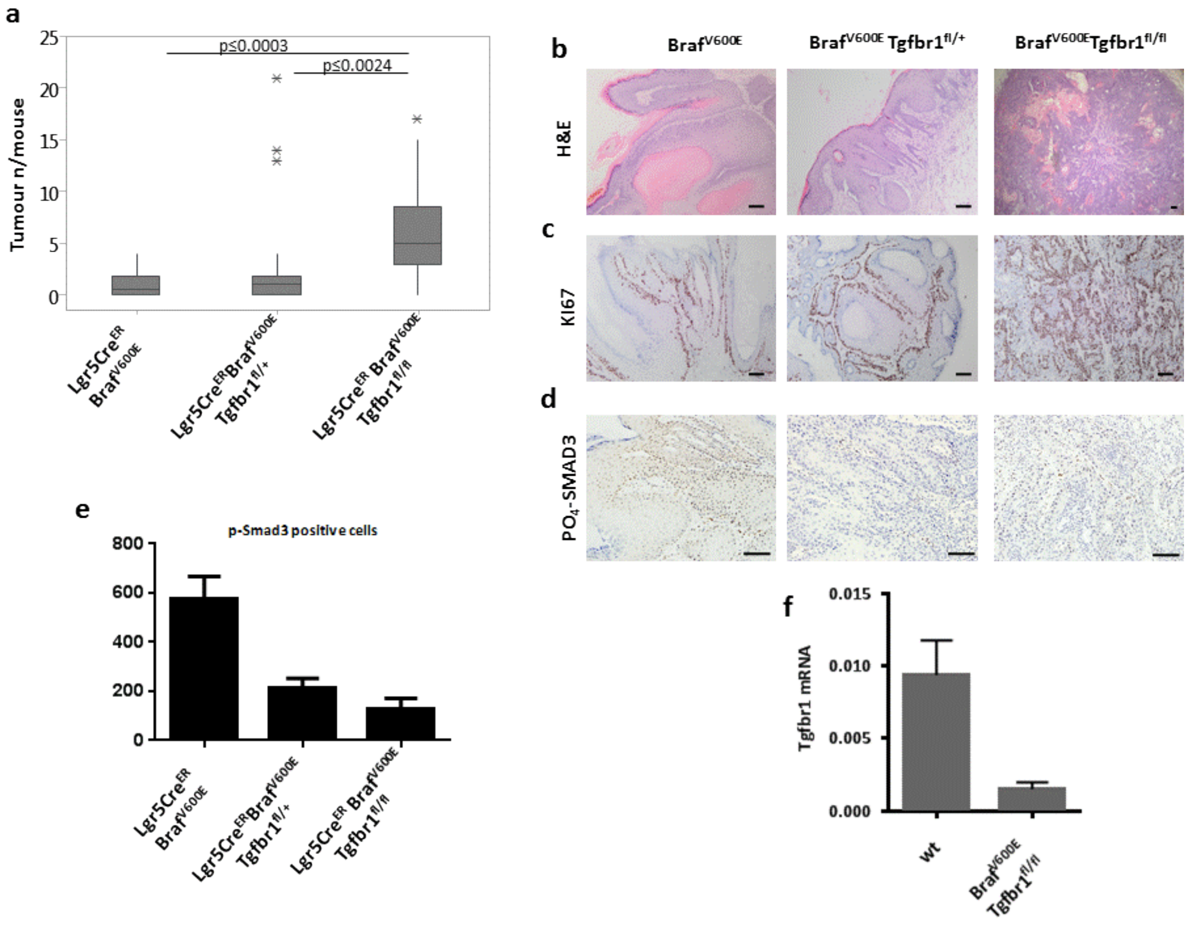

Supplementary Figure 12. **Loss of *Tgfbr1 along* with oncogenic *Braf*^{V600E} activation in LGR5^{+ve} cells leads to cSCC. (Related to Figure 7).** (**a**) Increasing tumor number with conditional deletion of *Tgfbr1*. Box and whisker plot of tumor number per mouse. *Lgr5Cre*^{ER} *Braf*^{V600E} (n=12), *Lgr5Cre*^{ER} *Braf*^{V600E} *Tgfbr1*$^{fl/+}$ (n=20) and *Lgr5Cre*^{ER} *Braf*^{V600E} *Tgfbr1*$^{fl/fl}$ (n=22). Mann-Whitney *U*-test was used to determine statistical significance. Whiskers donate the maximum and minimum; horizontal line donates median. * Outlier. Representative staining of (**b**) H&E, (**c**) KI67 and (**d**) PO_4-SMAD3 from tumors isolated from *Lgr5Cre*^{ER} *Braf*^{V600E}, *Lgr5Cre*^{ER} *Braf*^{V600E} *Tgfbr1*$^{fl/+}$ and *Lgr5Cre*^{ER} *Braf*^{V600E} *Tgfbr1*$^{fl/fl}$ mice. Scale bar, 100 µm. (**e**) Quantification of nuclear tumor cell PO_4-SMAD3 positive counted in 12 fields (magnification x200) per genotype (n=3 mice per genotype). Data are +/- s.e.m. (**f**) Q-RTPCR analysis of *Tgfbr1* mRNA from normal tissue (WT, n=4, biological replicates) and tumors (TUM, n=4, biological replicates) isolated from the indicated mice. Data are shown as ratios to the internal *Gapdh* control with error bars representing +/- s.e.m.

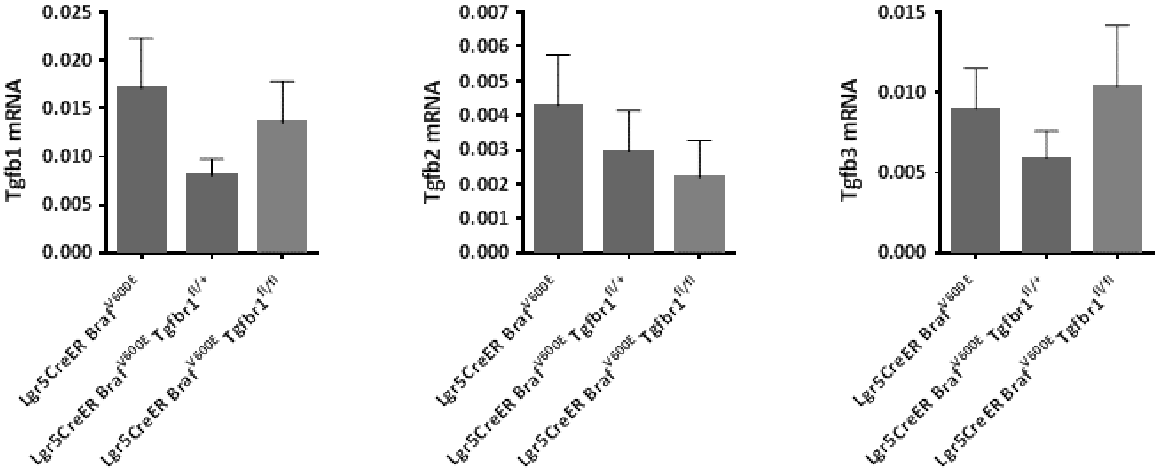

Supplementary Figure 13. Loss of *Tgfbr1* expression does not result in significant change in ligand mRNA expression. (**Related to Figure 7).** Q-RTPCR analysis for *Tgfb1*, *Tgfb2* and *Tgfb3* mRNA isolated from *Lgr5Cre*^{ER} *Braf*^{V600E} (n=4, biological replicates), *Lgr5Cre*^{ER} *Braf*^{V600E} *Tgfbr1*$^{fl/+}$ (n=3, biological replicates) and *Lgr5Cre*^{ER} *Braf*^{V600E} *Tgfbr1*$^{fl/fl}$ (n=4, biological replicates) tumors. Data are shown as ratios to the internal *Gapdh* control with error bars representing +/- s.e.m.

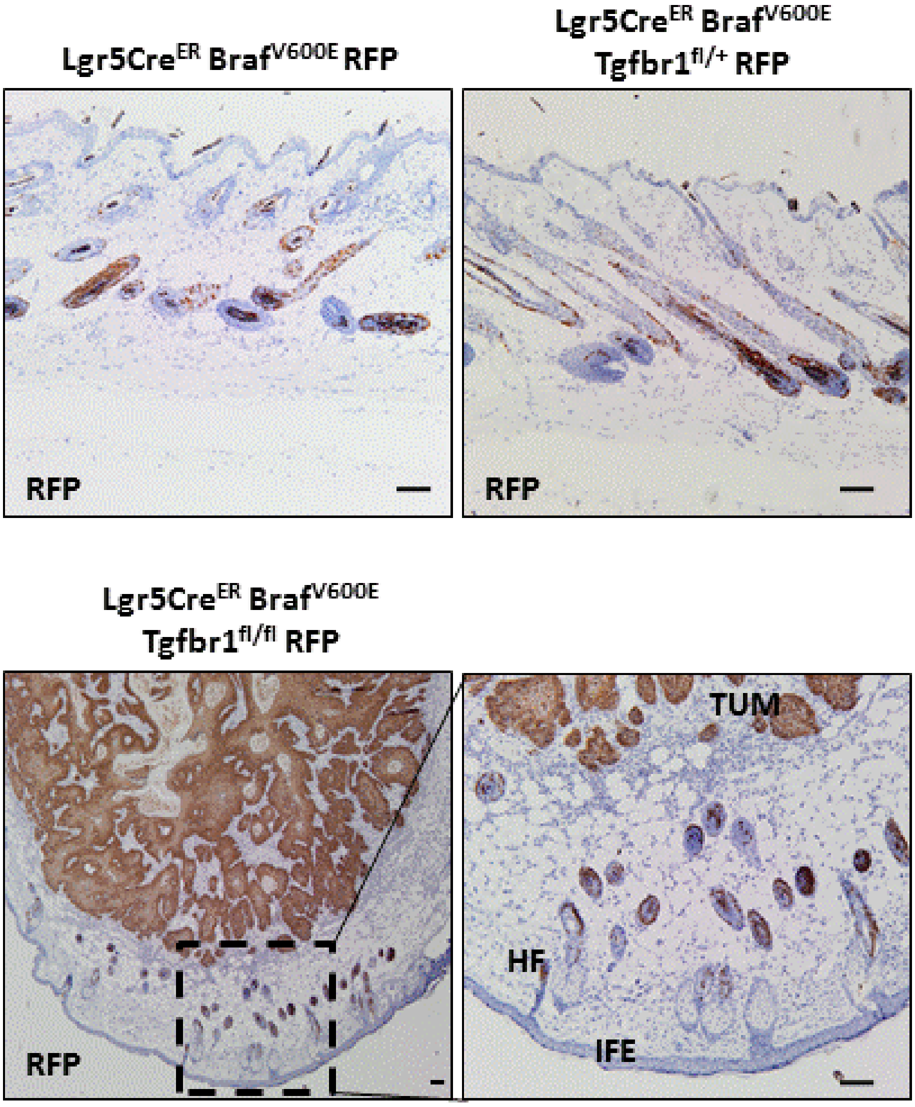

Supplementary Figure 14. Perturbation of TGFβ signaling is insufficient to disrupt skin compartmentalization. Representative images of RFP staining of *Lgr5Cre^{ER} Braf^{V600E}* (n=3), *Lgr5Cre^{ER} Braf^{V600E} Tgfbr1$^{fl/+}$* (n=9) and *Lgr5Cre^{ER} Braf^{V600E} Tgfbr1$^{fl/fl}$* (n=6) mice. Tumors are RFP positive. Inset shows region of normal skin above the tumor (TUM) with RFP staining in hair follicle (HF) bulge regions and absent from the interfollicular epidermis (IFE). Scale bar, 100 µm.

Figure 4 a:

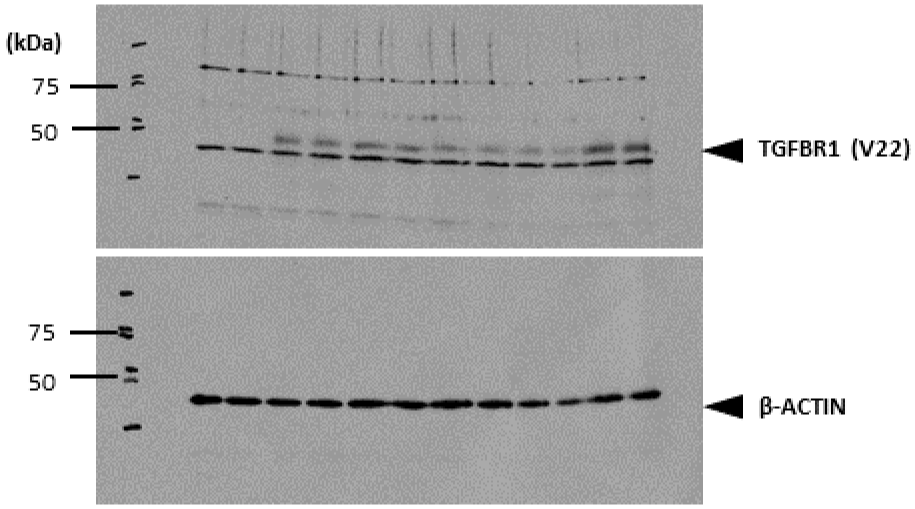

Figure 4 b:

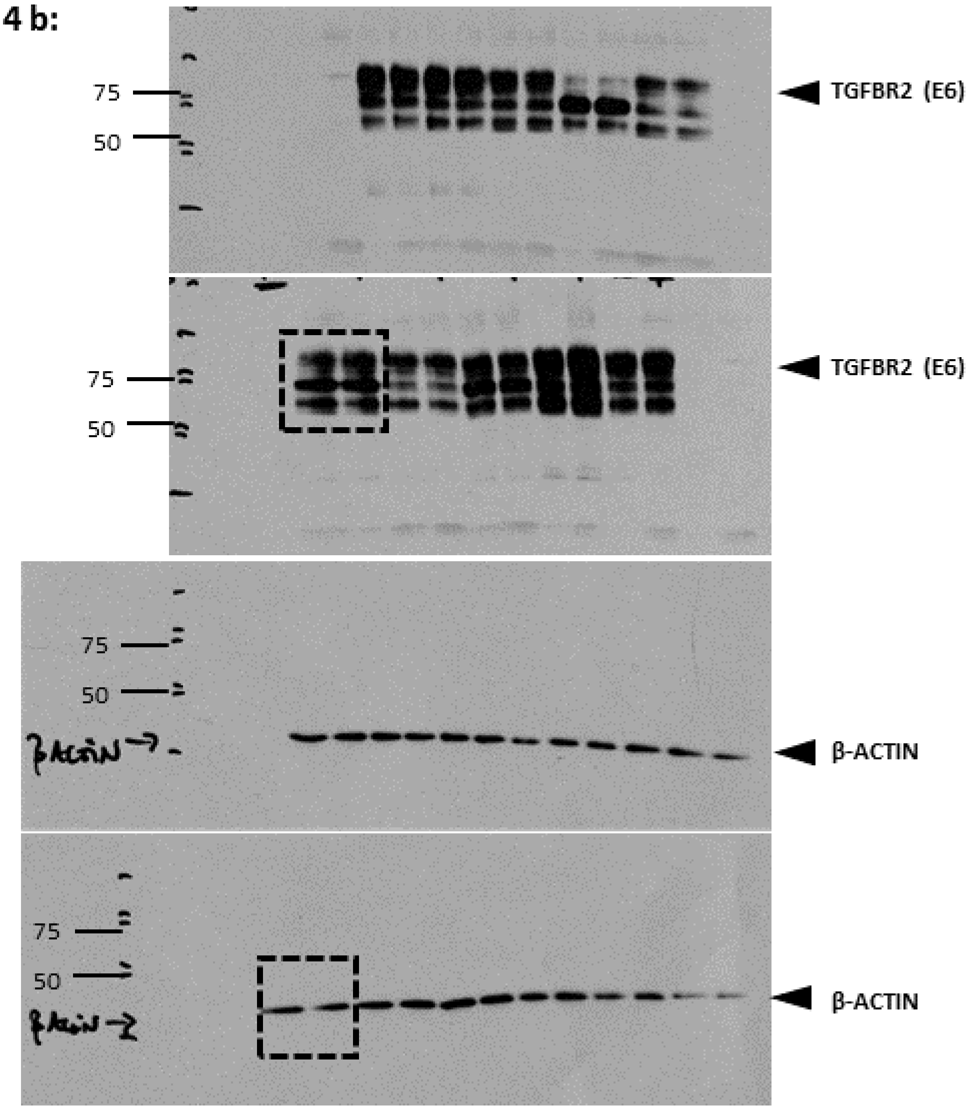

Supplementary Figure 5:

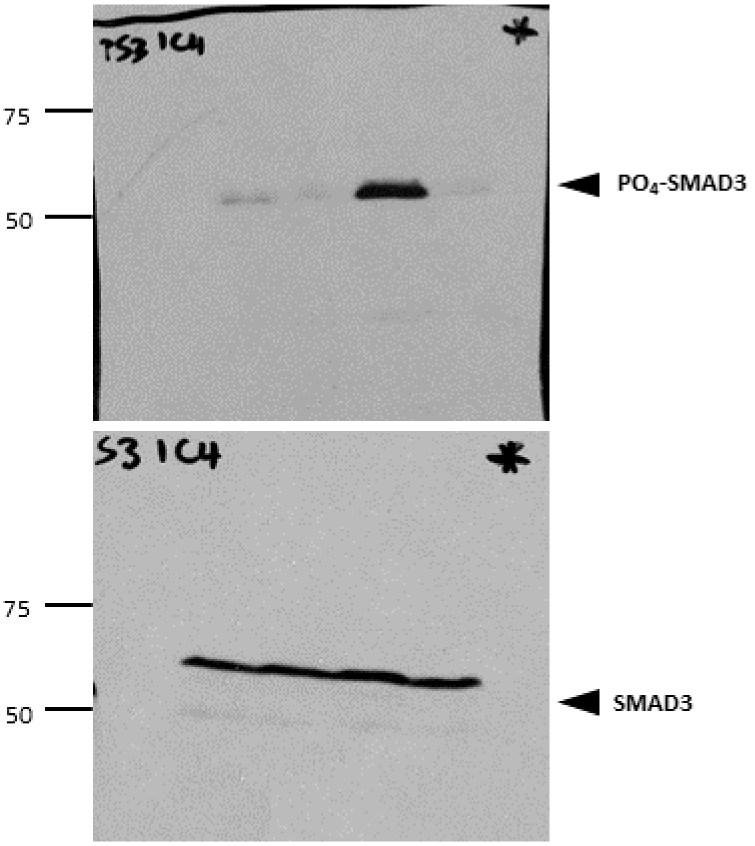

Supplementary Figure 7a:

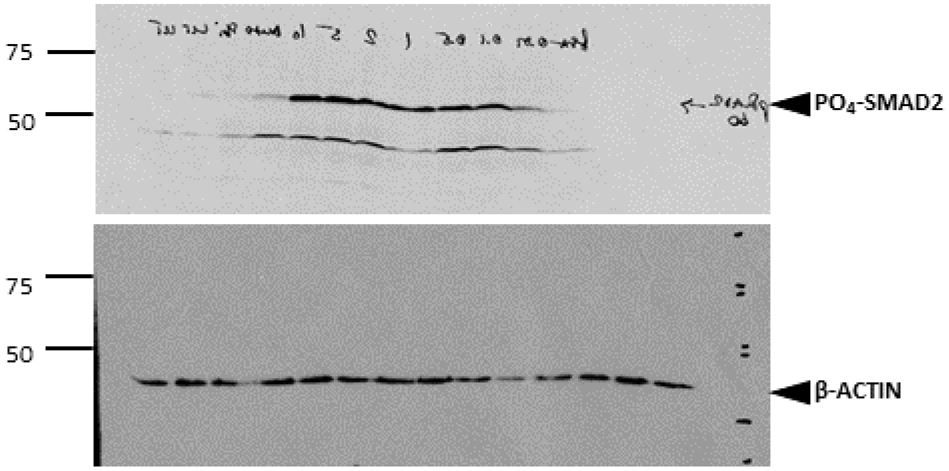

Supplementary Figure 7b:

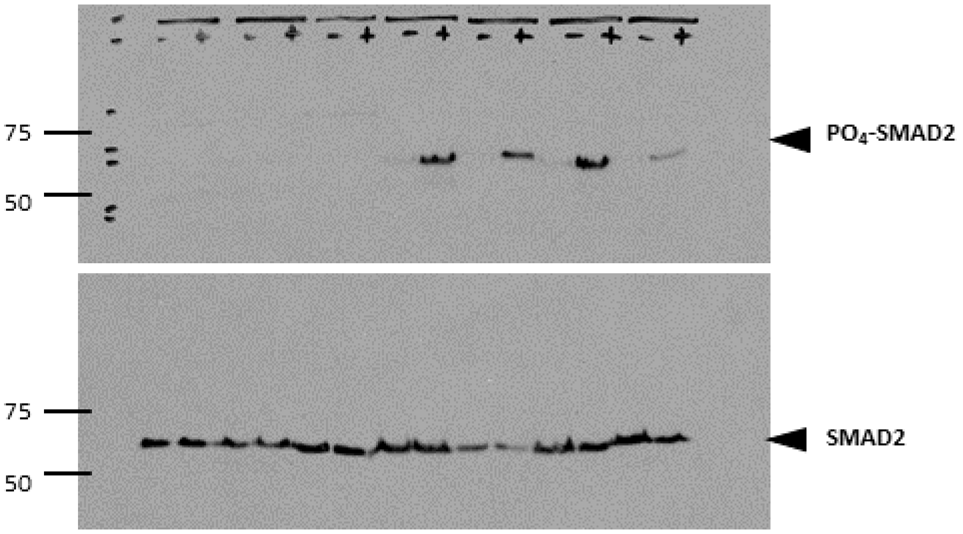

Supplementary Fig. 15. Uncropped original western blot scans.
Uncropped original western blot scans for Figure 4 (a-b), Figure 5 and Figures 7 (a-b).

Sample	Patient	Site	Lesion
V1	1	Right Parietal Scalp	Squamoproliferative
V2	1	Supra-pubic	Squamoproliferative
V3	1	Right Post-auricular	Squamoproliferative
V4	1	Arm	SCC
V5	1	Left Thigh	Squamoproliferative
V6	1	Left Back	SCC
V7	1	Right Shoulder	SCC
V8	1	Posterior Neck	Squamoproliferative
V9	1	Left Cheek	Squamoproliferative
V10	2	Chin	Squamoproliferative
V11	1	Left Parietal Scalp	Squamoproliferative
V12	1	Right Abdomen	Normal
V13	1	Lower back	SCC
V14	1	Left Popliteal Fossa	Squamoproliferative
V15	1	Left Post-auricular	Squamoproliferative
V16	1	Right Abdomen	Squamoproliferative
V17	1	Right Abdomen	Squamoproliferative
V18	1	Left chest	Squamoproliferative
V19	1	Left groin	SCC
V20	1	Right chest	SCC
V21	3	Left neck	Squamoproliferative
V22	3	Right Post-auricular	Squamoproliferative
V23	3	Left Arm	SCC
V24	3	Left Arm	Perilesional
V25	1	Right Arm	Perilesional
V26	1	Left Abdomen	Squamoproliferative
V27	1	Right Arm	SCC
V28	1	Left Abdomen	SCC
V29	2	Left Scapula	Squamoproliferative
V30	4	Abdomen	SCC
V31	1	Left Cheek	Squamoproliferative
V32	1	Left Upper Arm	Squamoproliferative
V33	5	Right Ear	Squamoproliferative
V34	5	Left Chin	Actinic Keratosis
V35	6	Right neck	SCC
V36	6	Right neck	Perilesional
V37	6	Right neck	Normal, sun exposed skin
V38	6	Left Shoulder	Squamoproliferative
V39	6	Left Lower Leg	Squamoproliferative
V40	6	Right Arm	SCC
V41	7	Right Neck	Normal, sun exposed skin
V42	7	Central Back	Squamoproliferative
V43	7	Upper back	Squamoproliferative
V44	7	Lower Back	Squamoproliferative
V45	7	Left Back	Actinic Keratosis

Supplementary Table 1. Vemurafenib-induced lesion details. Clinical characteristics of samples isolated from vemurafenib treated patients (adapted from South et al, 2014).

Patient	Site	Age	Sex	Immune Status
TGW1	chest	92	M	IC
TGW2	scalp	45	M	RT
TGW3	hand	65	M	RT
TGW4	hand	47	M	RT
TGW5	scalp	55	M	RT
TGW6	cheek	75	F	IC
TGW7	cheek	44	M	RT
TGW8	forearm	58	M	RT
TGW9	shin	55	M	RT
TGW10	hand	64	M	RT
TGW11	scalp	50	F	RT
TGW12	chest	73	F	RT
TGW13	scalp	52	F	RT
TGW14	hand	68	M	RT
TGW15	forearm	77	F	IC
TGW16	shoulder	61	F	RT
TGW17	forehead	75	F	IC
TGW18	temple	66	M	CT
TGW19	thumb	56	M	RT
TGW20	forehead	71	F	IC
TGW21	hand	77	F	RT
TGW22	hand	61	M	RT
TGW23	chest	56	M	RT
TGW24	forearm	51	M	RT
TGW25	hand	69	F	RT
TGW26	scalp	52	M	RT
TGW27	temple	60	M	CT
TGW28	nose	74	M	RT
TGW29	shoulder	67	M	CT
TGW30	forehead	67	M	IC
TGW31	chin	67	F	RT
TGM1	ear	57	M	RT
TGM2	neck	37	M	RT
TGM3	shoulder	65	M	RT
TGM4	scalp	82	M	IC
TGM5	forehead	77	M	IC
TGM6	temple	73	M	IS
TGM7	hand	58	F	RT
TGM8	hand	55	M	RT
TGM9	hand	68	M	RT
TGM10	shoulder	76	M	RT
TGM11	calf	84	F	IC
TGM12	nose	88	F	IC
TGM13	chest	92	M	IC
TGM14	hand	46	M	RT
TGM15	arm	55	M	RT
TGM16	ear	92	M	IC
TGM17	lip	34	F	RT
TGM18	scalp	66	M	IS
TGM19	shin	62	F	IS
TGM20	scalp	80	M	IC
TGM21	shoulder	63	M	RT
TGM22	nose	51	M	RT
TGM23	hand	78	M	IC
TGM24	shoulder	66	M	CT
TGM25	scalp	66	M	IC
TGM26	scalp	47	M	RT
TGM27	ear	71	M	IS
TGM28	shoulder	70	M	IC
TGM29	hand	91	F	IC
TGM30	leg	64	F	RT
TGM31	hand	77	M	IC
TGP1	ear	68	M	RT
TGP2	cheek	62	M	RT
TGP3	back	48	M	RT
TGP4	cheek	78	F	IC
TGP5	arm	71	M	IS
TGP6	chest	52	M	IS
TGP7	scalp	81	M	IC
TGP8	shin	88	F	IC
TGP9	back	57	M	RT
TGP10	scalp	83	M	IC
TGP11	forehead	54	M	RT
TGP12	cheek	91	M	IC
TGP13	nose	89	F	IC
TGP14	scalp	93	M	IC
TGP15	scalp	91	M	IC
TGP16	ear	83	M	IC
TGP17	scalp	86	M	IC
TGP18	leg	90	F	IC
TGP19	temple	78	F	IC
TGP20	scalp	92	M	IC
TGP21	scalp	67	M	RT
TGP22	forehead	75	M	IC
TGP23	hip	86	F	IC
TGP24	leg	90	F	IC
TGP25	ear	70	M	IC
TGP26	leg	100	F	IC
TGP27	forehead	93	M	IC
TGP28	scalp	96	M	IC
TGP29	scalp	83	M	IC

Supplementary Table 2. **Sporadic cSCC patient details.** Clinical characteristics of samples isolated from sporadic (non-vemurafenib induced) cutaneous SCC patients (adapted from South et al, 2014). IS = Immuno-suppressed, IC = Immuno-competent, RT = renal transplant, CT = cardiac transplant. M = Male, F = Female, TGW = Well Differentiated SCC, TGM = Moderately Differentiated SCC, TGP = Poorly Differentiated SCC.

Tumour	Age	Sex	Immune S	Site	Grade	Normal Skin	Tumour Type
TGW32	85	M	IC	Scalp	Well	Distant	Classic
TGM32	79	M	IC	Scalp	Mod	Distant	Classic
TGM33	89	M	IC	Scalp	Mod	Distant	Classic
TGM34	88	M	IC	Ear	Mod	Perilesional	Mixed Type
TGM35	88	M	IC	Scalp	Mod	Distant	Classic
TGP31	93	F	IC	Temple	Poor	Distant	Classic
TGP32	71	M	IC	Lip	Poor	Perilesional	Classic

Supplementary Table 3. **Sporadic cSCC patient details -Dundee Cohort.** Clinical characteristics of samples isolated from sporadic cSCC patients samples in Dundee. IC = Immuno-competent. M = Male, F = Female, Grade refers to differentiation status, well, moderate (Mod) or poor.

**Patient**	**Site**	**Age**	**Sex**	**Germline DNA**	**Immune Status**
PD_01	shoulder	71	M	lymphocytes	IS
PD_02	neck	83	M	normal skin, peri-lesional	IC
PD_03	wrist	64	M	lymphocytes	RT
PD_04	right neck	67	M	lymphocytes	RT
PD_05	ear	61	M	normal skin, foot	RT
PD_06	forehead	75	M	lymphocytes	IC
PD_07	scalp	65	M	lymphocytes	RT
PD_08	cheek	71	M	normal skin, sun exposed	RT
PD_09	chest	64	M	lymphocytes	RT
PD_10	ear	66	M	lymphocytes	RT
MD_01	shoulder	63	M	lymphocytes	RT
MD_02	forehead	72	M	lymphocytes	IS
MD_03	cheek	82	M	lymphocytes	RT
MD_04	hand	49	F	lymphocytes	RT
MD_05	arm	73	F	lymphocytes	RT
WD_01	jawline	64	M	lymphocytes	RT
WD_02	chest	80	M	lymphocytes	IC
WD_03	scalp	49	M	lymphocytes	RT
WD_04	forehead	65	M	lymphocytes	RT
WD_05	back	67	M	normal skin, non-sun exposed	CT
WD_06	hand	52	M	lymphocytes	RT
WD_07	leg	66	M	lymphocytes	RT
WD_08	ear	58	M	lymphocytes	RT
WD_09	dorsum hand	58	M	lymphocytes	RT
WD_10	leg	77	M	lymphocytes	IC
WD_11	chest	72	M	lymphocytes	RT
WD_12	scapula	64	M	lymphocytes	RT
WD_13	hand	68	M	lymphocytes	RT
WD_14	hand	75	F	lymphocytes	RT
WD_15	nose	74	M	lymphocytes	RT

Supplementary Table 4. Patient characteristics of 30 cSCC samples analysed by whole exome sequencing. Clinical characteristics of samples isolated from sporadic cutaneous SCC patients samples (adapted from and added to South et al, 2014). IS = Immuno-suppressed, IC = Immuno-competent, RT = renal transplant, CT = cardiac transplant. PD = poorly differentiated, MD = moderately differentiated, WD= well differentiated.
